# Optimising Strategies for *Plasmodium falciparum* Malaria Elimination in Cambodia: Primaquine, Mass Drug Administration and Artemisinin Resistance

**DOI:** 10.1371/journal.pone.0037166

**Published:** 2012-05-25

**Authors:** Richard J. Maude, Duong Socheat, Chea Nguon, Preap Saroth, Prak Dara, Guoqiao Li, Jianping Song, Shunmay Yeung, Arjen M. Dondorp, Nicholas P. Day, Nicholas J. White, Lisa J. White

**Affiliations:** 1 Mahidol-Oxford Tropical Medicine Research Unit, Faculty of Tropical Medicine, Mahidol University, Bangkok, Thailand; 2 Centre for Clinical Vaccinology and Tropical Medicine, Nuffield Department of Clinical Medicine, University of Oxford, Churchill Hospital, Oxford, United Kingdom; 3 Department of Infection and Tropical Medicine, Heartlands Hospital, Birmingham, United Kingdom; 4 National Center for Parasitology, Entomology and Malaria Control, Phnom Penh, Cambodia; 5 Kampot Provincial Health Department, Kampot, Cambodia; 6 Kampong Speu Provincial Health Department, Kampong Speu, Cambodia; 7 Research Center for Qinghao (Artemisia annual L.), Guangzhou University of Chinese Medicine, Guangzhou, China; 8 Department of Global Health and Development, Faculty of Public Health and Policy, London School of Hygiene and Tropical Medicine, London, United Kingdom; Kenya Medical Research Institute - Wellcome Trust Research Programme, Kenya

## Abstract

**Background:**

Malaria elimination requires a variety of approaches individually optimized for different transmission settings. A recent field study in an area of low seasonal transmission in South West Cambodia demonstrated dramatic reductions in malaria parasite prevalence following both mass drug administration (MDA) and high treatment coverage of symptomatic patients with artemisinin-piperaquine plus primaquine. This study employed multiple combined strategies and it was unclear what contribution each made to the reductions in malaria.

**Method and Findings:**

A mathematical model fitted to the trial results was used to assess the effects of the various components of these interventions, design optimal elimination strategies, and explore their interactions with artemisinin resistance, which has recently been discovered in Western Cambodia. The modelling indicated that most of the initial reduction of *P. falciparum* malaria resulted from MDA with artemisinin-piperaquine. The subsequent continued decline and near elimination resulted mainly from high coverage with artemisinin-piperaquine treatment. Both these strategies were more effective with the addition of primaquine. MDA with artemisinin combination therapy (ACT) increased the proportion of artemisinin resistant infections, although much less than treatment of symptomatic cases with ACT, and this increase was slowed by adding primaquine. Artemisinin resistance reduced the effectiveness of interventions using ACT when the prevalence of resistance was very high. The main results were robust to assumptions about primaquine action, and immunity.

**Conclusions:**

The key messages of these modelling results for policy makers were: high coverage with ACT treatment can produce a long-term reduction in malaria whereas the impact of MDA is generally only short-term; primaquine enhances the effect of ACT in eliminating malaria and reduces the increase in proportion of artemisinin resistant infections; parasite prevalence is a better surveillance measure for elimination programmes than numbers of symptomatic cases; combinations of interventions are most effective and sustained efforts are crucial for successful elimination.

## Introduction

Elimination of malaria from much of the world is a declared aim of the World Health Organization [1] and is currently being attempted or planned in many countries [2]. As the epidemiology of malaria varies widely, malaria elimination requires a variety of approaches individually optimized for different transmission settings. It is expensive and slow, or often impossible, to develop these approaches by trial and error in the field [3]. Mathematical modelling is a rapid, low cost means of using limited available data to compare large numbers of strategies and optimize their impact. It has great potential to help guide the efforts to achieve elimination [3]. Very little mechanistic modelling of malaria elimination has been attempted thus far [3]. One exception is models developed for malaria elimination in the context of newly discovered artemisinin resistance in Western Cambodia [4] for which mathematical modelling is helping to guide planning. Unfortunately, there are limited data on which to base models of malaria elimination using modern methods.

From 2004–2007, a large field study of malaria elimination using antimalarial drugs (termed ‘FEMSE’, Fast Elimination of Malaria by Source Eradication) in South-Western Cambodia was undertaken. Using mass drug administration and treatment with both artemisinin-piperaquine and primaquine the study was successful in reducing substantially the prevalence of malaria parasite positive individuals in most of the 26 villages studied, with elimination in 7 [5]. As in much of the region this was an area of low, unstable, seasonal, mostly forest fringe malaria transmission. The study was in two small areas of Kampot and Kampong Speu Provinces. The overall Annual Parasite Incidence (API) in 2004 in these provinces was around 6–8 confirmed cases per 1000 population per year [6], [7]. The results of this field study were in broad agreement with findings from previous mathematical modelling for Cambodia which showed that strategies that included high coverage of treatment with artemisinin combination therapies (ACT) can achieve large reductions over a similar timescale [4]. However, the malaria elimination field trial in South West Cambodia employed multiple strategies both simultaneously and sequentially and it was not known to what extent each strategy contributed to the successful outcomes. The execution of the trial varied geographically. This variation in the strategies employed between different sites, together with frequent monitoring of parasite rates, provided a range of data which could be used for fitting and validation of a mathematical model designed to answer specific questions about the trial.

These questions included the relative impact of mass drug administration versus augmented coverage of routine treatment (Rx) and whether adjunctive primaquine (PQ) in a single gametocytocidal dose was a worthwhile addition to either [8]. In the trial, primaquine MDA was given in a dose of 9 mg every 10 days for 6 months, an intervention that would be very resource intensive to replicate on a large scale. In two sub studies, an additional round of MDA was tried using ACT with single dose primaquine, one study at 42 days and another at 1 year. Large studies in the Comoros (32,519 subjects) [9] and Cambodia (28,143 subjects) [10] found mass administration of repeated low dose primaquine (9 mg) to be safe and well tolerated. Elsewhere, larger gametocytocidal doses of primaquine have been used [11], [12] and currently 0.75 mg/kg base as a single dose is recommended by the World Health Organization [13]. In a single dose, primaquine is currently under consideration for mass deployment in Cambodia [14] although there remains uncertainty over the optimum dose and benefit-risk ratio for this potentially haemolytic drug. Similarly a two day regimen was used for the ACT in the field trial as opposed to the more usual three days, also recommended by WHO [13]. The optimal dosing for these drugs is the subject of ongoing study.

There was concern, as in other areas, that reducing malaria prevalence will reduce population level immunity and a failed attempt at elimination might result in a subsequent rebound increase in malaria morbidity and mortality. The studied intervention was for three years following which malaria control measures were relaxed. Although the study population was screened for parasitaemia every 6 months, numbers of clinical cases were not recorded and active surveillance was discontinued at the end of the trial so inferences about changing population level immunity could not be made.

It was also not known if artemisinin resistance was present in the area during the study and what impact this may have had on the effectiveness of these strategies, or how these strategies may have affected the spread of drug resistance.

It is particularly urgent and important to answer these questions as ACT-based strategies are currently under consideration for malaria elimination throughout Cambodia [14] and in many similar countries worldwide and there is considerable ongoing debate about the possible impact of artemisinin resistance [4] and the potential role of primaquine [3], [8].

Detailed data from the Cambodian field study were used in combination with a range of other studies to develop and validate a mathematical model of *P. falciparum* malaria transmission for Cambodia. This model was used to answer a number of specific questions for the Cambodia National Malaria Control Programme to help with their planning of malaria elimination efforts. The broad aims were: 1. separate and quantitate the effects of the various components of the strategies used in the field study and predict their long-term impact; 2. explore interaction of these strategies with artemisinin resistance; and 3. design optimal elimination strategies. The results were distilled into five key implications for malaria elimination policy.

## Methods

**Figure 1:**
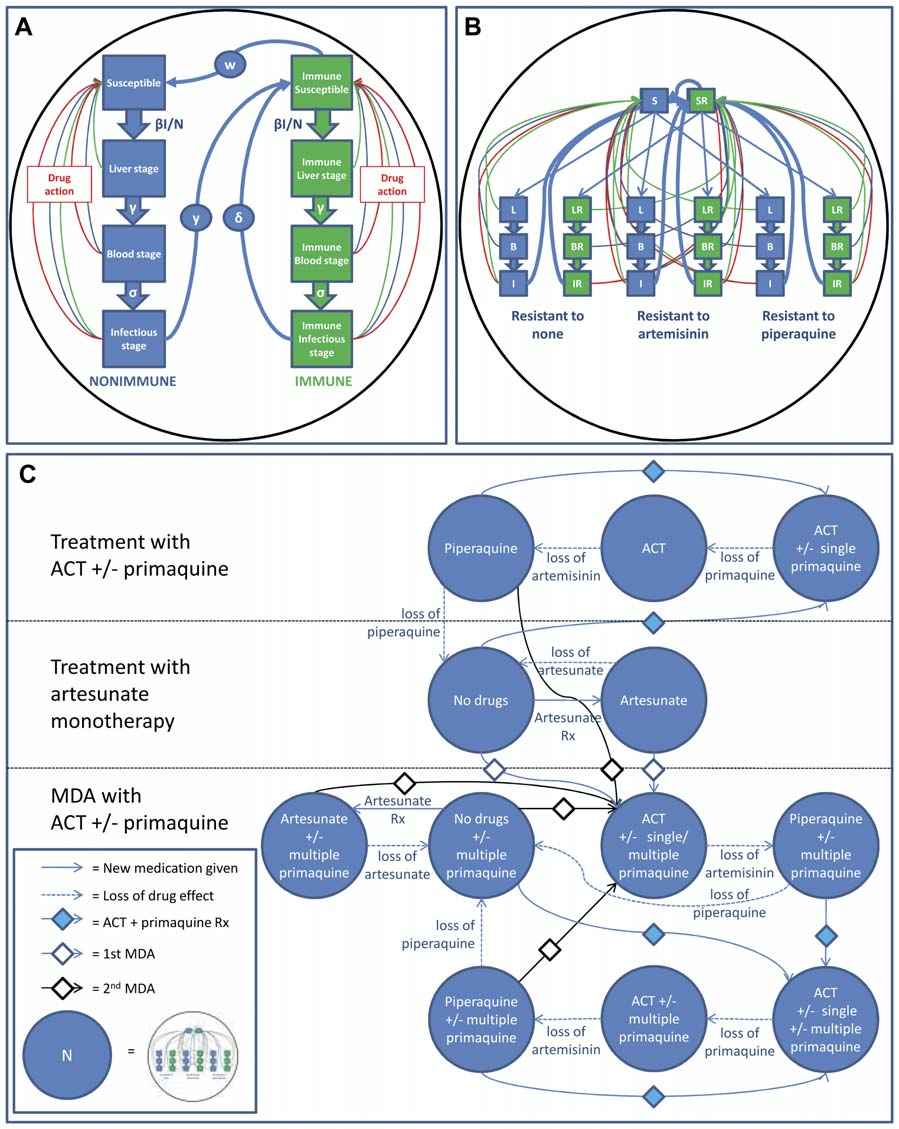
Summary of structure of mathematical model. **A** shows the basic model unit with parasite life cycle stages in the human host, antimalarial drug action and immunity. **B** shows the unit in **A** repeated three times to track parasites resistant to artemisinins and ACT partner drug. **C** shows multiple repetitions of **B** to reproduce the various strategies used in the trial. In A, ‘blood stage’ refers to individuals with asexual stage parasites in the peripheral blood but no gametocytes and ‘infectious stage’ is individuals with gametocytes.

A deterministic mathematical model of *P. falciparum* malaria transmission was developed using the Berkeley Madonnna™ software package (California, USA). The model structure is shown diagrammatically in figure 1 and as equations in Supporting Information S1. It incorporates stages of the *P. falciparum* life cycle in humans, antimalarial drug action, resistance to artemisinin and piperaquine, antidisease immunity, asymptomatic infection [15], births, non-disease deaths and details of the strategies employed in the trial. The basic framework was developed from a previously published model for artemisinin resistance [4] with major additions and modifications including the addition of host immunity and asymptomatic infections [15] using a method based on that of Aguas et al [16] and formal and extensive model fitting to, and validation with, malaria surveillance data and results from the field study (for details, see Supporting Information S2). Symptomatic infection was assumed to occur only in those with asexual parasites in the peripheral blood. Artemisinin resistance was modelled as previously using increased parasite clearance rates derived from field studies in Pailin, Cambodia [4], which first identified prolonged parasite clearance rates [17] although the prevalence was varied to explore its effect on the impact of the strategies under consideration. Parameters for malaria epidemiology were matched to those for the field study area and the strategies used in the trial were replicated in detail. Although similar reductions in parasite prevalence were found for *P. vivax* and *P. malariae* in the trial, only *P. falciparum* was modelled.

The modelled strategies were various combinations of:

- Treatment of symptomatic cases:
  ○artemisinin-piperaquine ACT
  ○adjunctive single dose of primaquine
- Mass drug administration:
  ○artemisinin-piperaquine ACT
  ○adjunctive single dose of primaquine
  ○multiple doses of primaquine: one dose given every 10 days for 6 months
- Long-lasting insecticide treated bed nets (LLITN)

ACT was given as a 2 day course of artemsinin-piperaquine (Artequick®, Guangzhou, People's Republic of China; 125 mg and 750 mg once a day respectively for adults) and each dose of primaquine was 9 mg. In the figures, for brevity, an adjunctive single dose of primaquine is referred to as ‘single primaquine’ and multiple doses of primaquine MDA as ‘multiple primaquine’.

Model Assumptions are listed in table S1. Model parameters and their sources are listed in table S2 with references in Supporting Information S3.

The fitted model was used to answer the following questions:

- What was the contribution of each component of the trialled strategies to the reductions in *P.falciparum* malaria burden?
- What will happen to the *P. falciparum* malaria burden (clinical cases and asymptomatic parasitaemic individuals) once these interventions are stopped at the end of the trial?
- What was the relative effect of the different strategies on population level immunity i.e. proportions of symptomatic versus asymptomatic cases?
- What would be the effect of artemisinin resistance on the effectiveness of these strategies and how do they affect its spread?
- What is the optimal design for an elimination strategy using these methods to achieve maximum long-term impact on *P. falciparum* malaria parasite prevalence?

## Results

### Fitting and validation of model with field data

**Figure 2:**
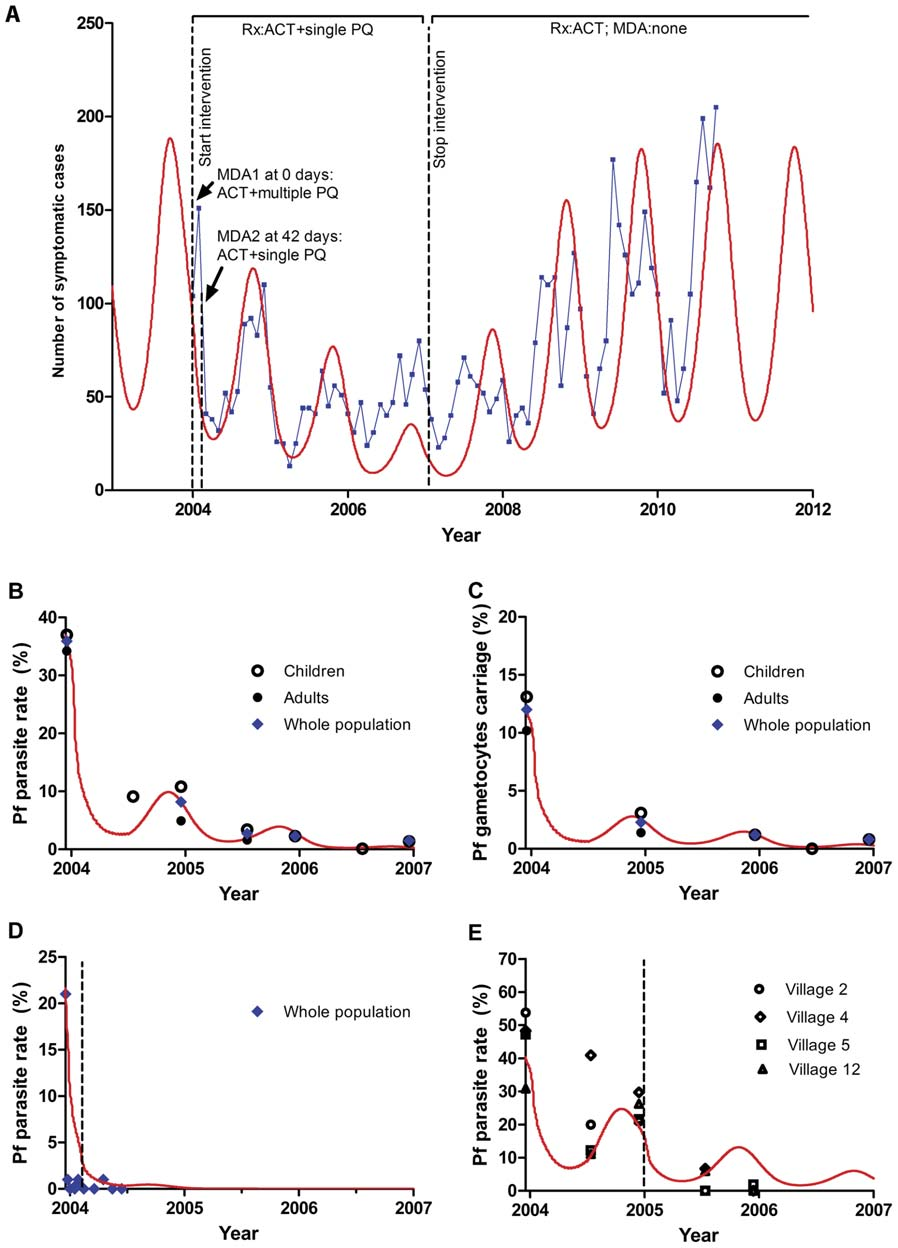
Model fits to data and validation. Model predictions are shown as red lines and surveillance data in black (subgroups) or blue (summary). **A** Model validated with to surveillance data for Kampot Operational District (OD) (2004–2010). The malaria control strategies used are shown. **B–E** Model fitted to data from the field study (2004–2007). **B–C** Reductions in *P. falciparum* asexual stage parasite (**B**) and gametocyte (**C**) prevalences in 17 villages in Kampong Speu OD. The strategy used was treatment with ACT plus single dose adjunctive primaquine for three years and a single MDA with ACT and multiple rounds of primaquine MDA. **D** Reduction in *P. falciparum* asexual parasite prevalence in 3 villages in Kampot OD with MDA and treatment as above, although with lower coverage, followed by a second MDA of ACT plus single dose adjunctive primaquine with higher coverage (dotted line) at 42 days. **E** Reduction in *P. falciparum* asexual parasite prevalence in 4 villages in Kampong Speu OD with the same strategy as **D** but with the second MDA (dotted line) at 1 year.

The model was able to reproduce closely malaria surveillance data from the national malaria control programme (figure 2A) and the results of the study (figure 2B–E) with realistic values for coverage with the various components of the strategies employed. Further details of the fitting and validation are given in the supporting information with derived coverages for the various components of the interventions shown in table S3.

### Analysis

#### Contribution of each component

**Figure 3:**
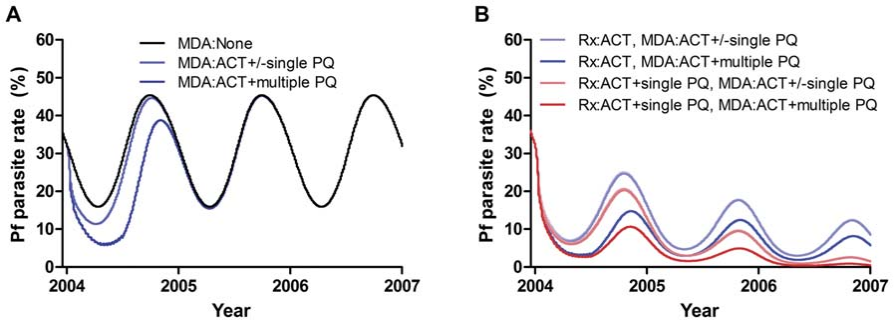
Contribution of each component of the strategies employed on *P. falciparum* overall parasite prevalence. Each panel shows the additional effects of adding primaquine to MDA with ACT. **A** MDA alone and **B** MDA combined with simultaneous introduction of ACT plus or minus single dose adjunctive primaquine for treatment. Black line is no treatment, blue lines are no treatment (A) or treatment with ACT (B), red/pink lines are treatment with ACT plus primaquine.

Figure 3 shows the modelled effect of each component of the elimination strategies employed in the study using the coverages of 78% for treatment and 95% for MDA derived from fitting the model to data (table S3). Results for gametocyte carriage are shown in figure S1.

The model showed that MDA produced most of the initial reduction in parasite prevalence. ACT MDA reduced parasite prevalence by 26% and the addition of multiple rounds of primaquine MDA increased this to 65% (figure 3A), whereas adding a single dose of primaquine to ACT MDA had <1% additional affect. Timing of the primaquine dose simultaneously with the ACT was partly responsible for this limited effect observed in the model, and later doses had more effect. Introducing ACT treatment without MDA produced an initial drop in parasite prevalence of 23%, less than that due to MDA alone at the same coverage. Changing treatment to ACT plus single dose primaquine added a further 20% initial drop in prevalence to that due to MDA. MDA alone at an estimated coverage of 95% was insufficient to achieve elimination (defined as <1 malaria parasitaemic individual), producing only a temporary reduction in the number of cases lasting under 1 year following which the parasite prevalence returned to the pre-intervention equilibrium. This was because the timing of the MDA was not optimal (see below), coverage and adherence were not 100% and it was not logistically feasible to provide MDA to the whole population simultaneously. Transmission occurred from infected people who had not yet received MDA and even with repeated rounds of MDA, there was ongoing transmission in the time between rounds.

The long-term decrease in prevalence was mostly due to the introduction and continuation for 3 years of high coverage ACT for treatment of people with fever (figure 3B). This effect was significantly enhanced (10–13% additional decrease in parasite prevalence) by the addition of a single dose of primaquine treatment. In the field study the high coverages were achieved by use of trained village malaria workers and a high profile advertising strategy.

Sensitivity analyses found the relative effects of different strategies to be robust to changes in coverage for the interventions, duration of immunity and the proportion of immune patients who became symptomatic. Changes in immunity did not alter the effect of MDA but did change the impact of treatment. Varying the duration of immunity from 1 day to 5 years produced a diminishing decrease in the size of the initial drop in parasite prevalence due to introducing high coverage with ACT treatment from 34% to 13%. Varying the proportion of immune patients who became symptomatic from 0–100% changed the initial drop in prevalence resulting from this ACT treatment from 13–34%.

The predicted time to elimination (<1 parasitaemic individual) for the main study intervention of a combination of simultaneous introduction of high coverage with ACT plus single dose primaquine for treatment and MDA with ACT plus multiple doses of primaquine every 10 days for 6 months was 4.2 years. A second round of single dose MDA made little difference to this result. Without primaquine treatment, continued use of high coverage ACT treatment after MDA with multiple doses of primaquine eliminated malaria more slowly over 7.3 years. These times are relatively long because of the high baseline parasite prevalence in the study population and incomplete adherence to the medication (assumed for this study to be 77%).

The predicted times to elimination were affected greatly by parameter values used for immunity despite immunity having no direct effect on transmissibility in the model. This was because immunity affected the proportion of cases that became symptomatic and were treated. Assuming 10% of immune patients were symptomatic, changing the duration of immunity between e.g. 1 day, 1, 2 and 3 years increased the time to elimination exponentially from 1.2, 4.1, 9.2 and 23.2 years and longer durations of immunity precluded elimination. If 20% of immune patients were symptomatic, these times were reduced to 1.1, 3.3, 6.1 and 9.2 years with elimination in 21.3 years with immunity lasting 5 years. Greater percentages of immune patients being symptomatic further reduced the times to elimination as more of them received treatment.

The results were robust to altering the parameters for primaquine efficacy. The effect of low dose primaquine (9 mg) on liver stage parasites is unknown and probably very small. Varying the rates of clearance of liver stage parasites due to primaquine in the model made no noticeable difference to the relative effect of strategies including primaquine or the times to elimination. The efficacy of this low dose of primaquine on gametocytes is also uncertain, although known to be significant at larger doses. Its effectiveness was thus varied from that estimated for 0.75 mg/kg (see table S2) to an effect 4 times smaller. Large reductions in primaquine efficacy against gametocytes were required to significantly alter the results. When the effectiveness of primaquine against gametocytes or the coverage of primaquine was halved, the time to elimination for ACT plus primaquine MDA and treatment increased from 4.2 to around 5.2 years and, when halved again, to 7.2 years.

#### Cessation before elimination

**Figure 4:**
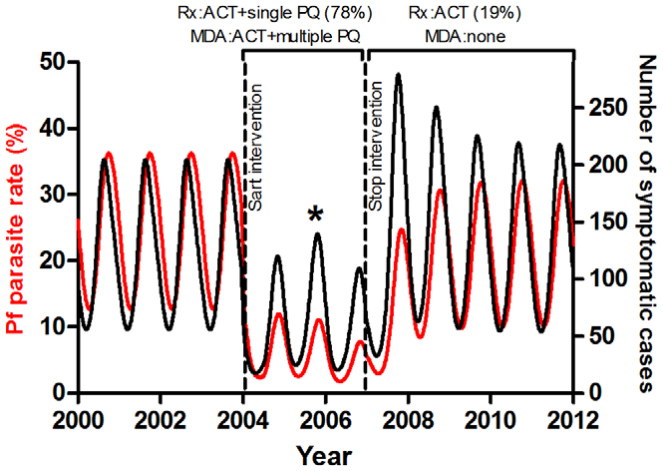
Population level immunity. Predicted numbers of symptomatic cases and proportion with parasites in the blood before, during and after the trial. The red line is the percent of the population with blood stage parasites and the black line is the number of symptomatic cases. The * indicates the paradoxical increase in clinical cases despite a decrease in the proportion affected.

The model was used to predict what might happen when the study ended in 2007 if funds were not available to continue to provide ACT+single dose primaquine treatment at high coverage. To simulate this, primaquine was stopped and coverage with ACT treatment reduced from 78% to 19% at this time (figure 4). These coverages were derived from fitting the model to surveillance and trial data. The model predicted a steady increase in parasite prevalence over the following three years to a new equilibrium level. This would be the scenario if an elimination effort had to stop before total elimination had been achieved because of insufficient long-term funding or policy changes.

#### Population level immunity

The field study demonstrated a fall in the prevalence of *P. falciparum* parasitaemia over the three year study period but did not collect data on the numbers of symptomatic malaria cases. Similar reductions in parasite rates were found for *P. vivax* and P. *malariae*, although these were not modelled. The model indicated that numbers of symptomatic cases do not mirror numbers of people with parasites because of changing levels of immunity which protect people from symptomatic infection. Rather, falling immunity leads to an increase in the proportion that is symptomatic. The model also predicted that with a successful elimination strategy, as numbers of parasitaemic people continue to fall, numbers of symptomatic cases can level off or even increase as population level immunity declines (figure 4). Following the end of the field study, the model predicted an initial large increase in symptomatic cases to numbers greater than before the study period despite malaria parasite prevalence being lower. This was because of the reduction in population level immunity during the intervention when there were fewer infections.

To assess the sensitivity of these results to the duration of immunity, its mean value was varied from 0.5–5 years. The longer lasting the immunity, the lower the number and proportion of symptomatic cases. In addition, a longer duration of immunity reduced the relative impact of increasing coverage with treatment but not the other interventions. This was because it increased the proportion of asymptomatic cases. Even with a duration of immunity of 5 years, however, a high coverage with ACT treatment was still the most effective strategy in the long-term. The other results reported above were robust to this changing duration of immunity. Increasing the percentage of immune patients who became symptomatic delayed the increase in both symptomatic and asymptomatic infections upon cessation of the intervention because their numbers fell further during the intervention as a higher proportion was treated.

#### Artemisinin resistance

**Figure 5:**
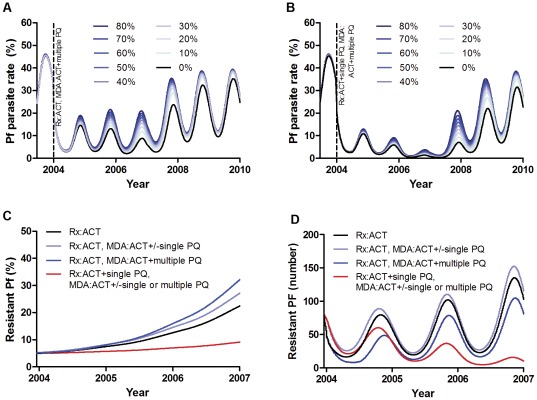
Artemisinin resistance and different elimination strategies. Effect of an increasing prevalence of resistance (defined as the proportion of infections which are artemisinin resistant) from 0% to 80% on the success of MDA with ACT plus multiple rounds of primaquine and treatment with **A** ACT or **B** ACT plus single dose primaquine from 2004–2007. **C–D** effects of different treatment and MDA regimes on the spread of artemisinin resistance (**C**: prevalence of resistant infections, **D**: number of resistant infections), presuming a starting prevalence of resistance of 5%. Interventions ceased in 2007.

In the model, the effectiveness of treatment and MDA with ACT on parasite prevalence was reduced by increasing the prevalence of artemisinin resistance (figure 5). In these simulations, the effectiveness of ACT waned over time as the resistant infections spread more quickly in the presence of continuing high coverages with ACT treatment. Once coverage with ACT treatment fell at the end of the trial, both artemisinin sensitive and resistant infections increased and the resistant proportion increased more slowly as the selection pressure was reduced. Figure 5A shows the effect of artemisinin resistance on the impact of treatment with ACT plus MDA with ACT and multiple rounds of primaquine. The addition of Primaquine to each treatment (figure 5B) largely negated this effect.

Compared to before the trial when malaria treatment in the study area was thought to have comprised a variety of non-artemisinin antimalarials and low level artemisinin monotherapy, the proportion and number of artemisinin resistant infections increased much more quickly when ACT was introduced for treatment and only slightly more when MDA was added as well (figure 5C). Single dose primaquine added to ACT treatment greatly slowed this increase in artemisinin resistance. This was because it reduced transmission of resistant parasites sufficiently to prevent epidemic behaviour of the resistant subpopulation.

Multiple rounds of primaquine MDA greatly decreased the number of resistant infections whereas a single round of primaquine MDA did not (5D). The proportion of resistant infections was unaffected by a single primaquine MDA but increased slightly when multiple rounds of primaquine MDA were added (5C). This apparent paradoxical increase was because primaquine further reduced the number of infections and thus population level immunity. Infections could then spread more rapidly in the population, although only resistant infections increased in number as selection pressure from ACT continued to reduce the number of sensitive infections. In contrast, the effect of adding primaquine to treatment was cumulative over the course of the study and this ongoing additional effect was sufficient to prevent the spread of both sensitive and resistant infections. This was robust to varying the duration of immunity between 0.5 and 5 years.

### Optimization and design of future studies

The model was used to design optimal elimination strategies for testing in future field studies. This was done by varying timing of the different components, combining different interventions and investigating the effect of adding new interventions that were not in the field study, e.g. transmission blocking by insecticide treated bed nets.

#### Timing

**Figure 6:**
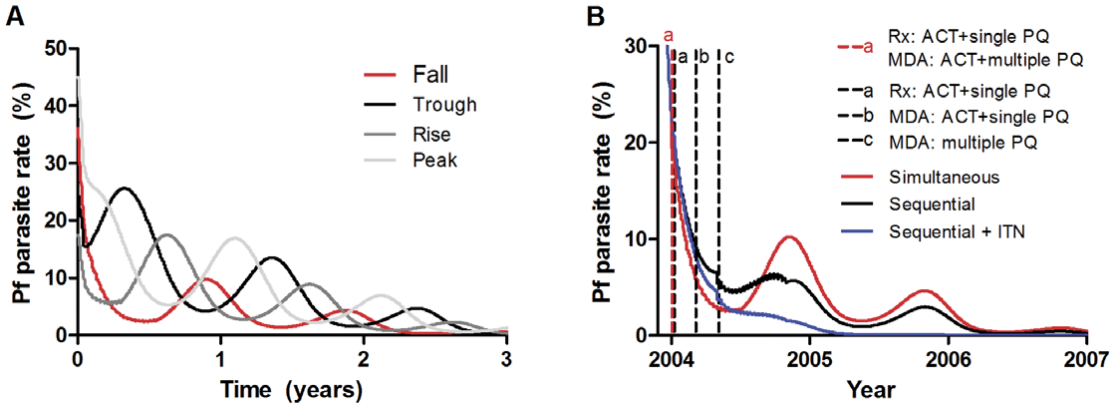
Effect of varying the timing of interventions. **A** Varying the season in which interventions are implemented. MDA with ACT+multiple primaquine and Rx with ACT+single primaquine started together at different times during the malaria season: when parasite prevalence is falling, at trough, rising or at peak. **B** Simultaneous versus sequential interventions and the additional effect of bed nets. Simultaneous introduction of treatment with ACT+single primaquine *plus* MDA with ACT+multiple primaquine (red) versus optimally timed sequential interventions (black): a. treatment with ACT+single primaquine then b. MDA with ACT+primaquine at 9 weeks before the nadir and c. another MDA with multiple primaquine at the nadir and the same simultaneous interventions with the addition of long lasting insecticide treated bed nets with 30% coverage (blue).

There were no significant differences in the long-term rates of decline or times to elimination when interventions were introduced together at different times of year (figure 6A). Although combined strategies which included MDA caused a predicted greater initial decline in parasite prevalence if introduced when seasonal malaria was not at peak prevalence, this difference was not maintained. This is because the impact of the MDA was short-lived. There was thus no clear optimal time for simultaneous introduction of MDA plus treatment (as was done in the trial, figure S2A). When multiple interventions were introduced in the model at different times, however, the relative timing of each intervention became important, as outlined below.

#### Combining Interventions

Combinations of interventions were predicted by the model to be much more effective than single interventions. This was particularly true when combining strategies which act at different parts of the parasite life cycle e.g. ACT against blood-borne parasites and transmission-blocking measures against mosquitoes, specifically long-lasting insecticide treated bed nets. The effect of multiple interventions using multiple different antimalarial drugs was greatest when they were introduced at different times (figure 6B). The optimal time for a single round of MDA was from 6 to 12 weeks before the nadir of seasonal parasite prevalence (figure S2B). In the field study, the MDA was approximately 14 weeks before the nadir. Where two or three rounds of MDA were used in the model, they had maximal impact if the final round of MDA was completed before the nadir e.g. one round each month for three months. When doing this, 3 rounds of ACT MDA over 3 months was found to be optimal and of comparable efficacy to the 19 rounds of primaquine MDA over 6 months used in the trial. MDA with multiple rounds of primaquine was most effective after a single round of ACT MDA when started at trough parasite prevalence. These optimal timings were because the effects of the different strategies overlapped and with shorter intervals the effects of the additional benefit from each strategy were reduced.

#### Transmission blocking

Insecticide treated bed nets were predicted to have a relatively large additional impact whenever they were introduced in addition to antimalarials. The optimal time was as early as possible, regardless of season (figure 6B). This was despite assuming only 30% efficacy in reducing transmission and was robust to changes in coverage from 50–100%. This large additional effect of bed nets was true for all strategies due to their transmission-blocking action at a different stage of the parasite life cycle from antimalarials. Bed nets alone, however, were never sufficient to eliminate malaria and combination with other interventions was always required to achieve this.

#### Optimal elimination strategy

From this model can be derived an optimum strategy for malaria elimination in this context. The most robust modelling results were that ACT treatment with high coverage is an essential component without which elimination cannot be achieved. If continued for long enough at high coverage, this alone may be sufficient. Adding adjunctive primaquine to ACT treatment accelerated elimination and adding other interventions (MDA and LLITNs) further accelerated the process. To achieve maximal impact, MDA should be used as levels of infection fall in the low season and multiple rounds of MDA should be completed before the seasonal nadir in parasite prevalence. Three rounds of ACT MDA over three months were found to be optimal with little advantage from adding primaquine MDA.

## Discussion

Mathematical modelling using data from a recent field trial in Cambodia showed that the major contributor to the large reductions in parasite prevalence seen over 3 years was use of high coverage with artemether-piperaquine ACT for treatment of fever cases. In contrast, MDA with this ACT produced a large initial drop in infected people but its effect was not sustained beyond 1 year. This was primarily because the MDA was only employed for a short period and the number of infections increased again after it ceased whereas artemether-piperaquine ACT treatment continued for the entire 3 year study period. Although the initial effect of MDA was greater than that of treatment, the cumulative effect of longer availability of ACT treatment was greater in the long-term.

From field studies, it is not clear to what extent 9 mg of primaquine affects gametocytes and reduces malaria transmission. The effect of a higher single dose of adjunctive primaquine [13] carries a greater risk of haemolysis but its gametocytocidal effect may be greater. To achieve the best fits of the model to field data required the inclusion of an effect of a similar magnitude to 0.75 mg/kg. When this was included in the model, the effect of ACT used for treatment was significantly enhanced by the addition of a single dose of primaquine, as recently found in a field study in Myanmar, using 0.75 mg/kg [11]. These findings provide support for large scale deployment of primaquine to help eliminate malaria but highlight the need for dose ranging studies. Primaquine is now being considered for country-wide use in Cambodia as an adjunct to ACT with the aim of elimination [18].

For primaquine MDA, the modelling results were less encouraging. The modelling indicated that adding a single round of primaquine MDA had very little impact on numbers of infections, and the cumulative effect of multiple rounds would be required. Multiple repeated doses of primaquine given as MDA more than doubled the initial effect of the ACT MDA in the model. This would be impractical and costly to implement on a large scale. A more pragmatic strategy would be to do several rounds of MDA, one every few weeks. Three rounds of MDA with ACT were found to be optimal in the model and most effective if completed in the 3 months before the nadir in parasite prevalence.

As well as uncertainties about the effective dose, there remains another important barrier to the rollout of primaquine. It can cause dangerous haemolysis in people with certain types of a common genetic abnormality, Glucose-6-phosphate dehydrogenase (G6PD) deficiency. This effect is thought to be dose related and administration of a single low dose, as was used in this trial, should minimize this. In the modelled field study, no cases of significant haemolysis were reported. Previous studies in Cambodia and Myanmar with 9 mg and 0.75 mg/kg base respectively did not encounter problems with haemolysis [10], [11]. It is not certain how prevalent G6PD deficiency is in Cambodia (estimates range from 15–20%, the most common variant being G6PD Viangchan (871G>A) [19], [20]) and field studies are currently underway to investigate this.

The model was used to predict changes in immunity and symptomatic cases after the end of the trial. Immunity in malaria remains poorly understood; this is an important limitation for any model of malaria. We thus chose a relatively simple structure for immunity that has been previously validated for a range of transmissions settings. We also varied the parameters used for immunity within wide ranges to check the robustness of the results.

In the model, when ongoing high coverage with ACT and single dose primaquine treatment was stopped, mimicking cessation of the project, a subsequent steady rise in parasite prevalence was predicted. In addition, due to a decrease in the population level immunity, the number of clinical cases initially increased to levels greater than before the intervention. This was despite continued availability of ACT at a lower rate of coverage. This would be the situation if a short-term elimination strategy were attempted and then funding withdrawn when numbers of infections were low. This, combined with the long-term decrease being primarily due to the ACT treatment illustrate the importance of ensuring sufficient long-term funding is available for elimination programmes. Even with a successful ongoing malaria elimination strategy, in some situations, numbers of symptomatic cases in the model were seen to increase. This was because as numbers of infected people fell, population immunity decreased and the proportion of people who were symptomatic increased. In the initial stages of an elimination strategy this can result in a levelling off or even increase in the number of symptomatic cases, although this will subsequently fall as numbers of infected people continue to decline [15]. This phenomenon has been observed in the field [21], [22], although it has not been possible to separate the role of declining immunity from other possible contributors. Regardless, the cumulative number of clinical cases following an intervention is always predicted by the model to be lower than if there had been no intervention. Where this paradoxical effect occurs, an intervention may appear falsely to be failing. Malaria surveillance systems typically rely on numbers of reported cases in the absence of data on asymptomatic parasitaemia (passive surveillance) and this finding highlights the importance of regular population screening for overall parasite prevalence to capture both the symptomatic and asymptomatic cases [23], to get a more accurate picture of the effectiveness or otherwise of elimination strategies. When the intervention was stopped in the model, numbers of symptomatic cases increased more rapidly than overall numbers of parasitaemic people due to a lack of immunity in the population following the period of low prevalence. In this case, although obviously undesirable, this rapid rise may be beneficial as an early warning sign of increasing parasite prevalence.

This modelling exercise showed that to have a realistic chance of eliminating malaria from an area, a combination of different strategies is required. MDA can significantly reduce the number of infections in the short term (in this study <1 year), particularly when repeated in the low transmission season, but high coverage with ACT is also required for a prolonged period (4–7 years in this study). Elimination is greatly accelerated by the co-deployment of long-lasting insecticide treated bed nets and further enhanced by the transmission blocking effect of adjunctive primaquine treatment. All this requires significant investment including in longer-term initiatives such as training large numbers of village malaria workers to provide high coverage ACT in remote areas. This is the current strategy adopted by Cambodia which is embarking on a massive scale up of the village malaria worker scheme with funding from the Global Fund to Fight Aids, Tuberculosis and Malaria.

One potential spanner in the works that has not been considered in this model is population migration. In-migration of infected individuals reintroduces infection which can make elimination significantly more challenging. There are little data on migration of different population groups in many malaria endemic countries, including Cambodia, and the degree of risk to any future elimination programme is uncertain. Attempts are being made in Cambodia to address this issue and studies are underway to quantify and characterize population migration. If in-migration of infected individuals is a significant contributor to malaria transmission in Cambodia these migrant groups must somehow be included in any elimination strategy, most likely using a similar combined approach to that outlined above.

Another potential limitation of this model was the assumption that only a single infection occurs at any one time in an individual. Although usually true in low transmission settings [24], this is not the case where transmission is high. It is not known how such multiple clones would interact and affect the transmission dynamics thus it is difficult to predict how they may impact on the results presented (Table S1). For this to be modelled realistically, further clinical and laboratory research is needed.

A reassuring finding from the model was that artemisinin resistance, in its current mild form [17], does not appear to have a large impact on the effectiveness of the regimes used in the trial for elimination. This was true even with the current highest estimate of 10% of infections being resistant [24]. In the presence of hypothetical very high modelled prevalences of artemisinin resistance (70–80%), the effect of ACT was clearly diminished but the addition of primaquine to the ACT largely negated this reduction. As in a previous model of artemisinin resistance [4], ACT accelerated the increase in the number and proportion of artemisinin resistant infections, despite greatly decreasing the number of artemisinin sensitive infections. This was worsened by the addition of ACT MDA. This increase in resistance was significantly slowed by the addition of primaquine to ACT treatment as it had sufficient antimalarial action to decrease the overall numbers of both resistant and sensitive infections. These findings may be different if the current mildly resistant phenotype changes to be more resistant, although this was not included here as it is known what form this phenotype may take.

The model was used to explore a number of scenarios that were not included in the original field study. This was in order to explore possible means of optimizing the strategies used in order to assist with planning future field studies. Using modelling this way can be much more rapid and efficient than trialling multiple variants of strategies in the field. The modelling indicated that multiple combined interventions are more effective than single interventions and it is preferable to use different interventions which impact on the same part of the parasite life-cycle sequentially rather than simultaneously to maximize their long-term impact. This is presumably because there is a maximum effect for drugs with a similar mechanism of action and additional drugs will have no additional impact beyond this maximum. The addition of long-lasting insecticide treated bed nets to any strategy greatly enhanced its effectiveness, despite assuming low efficacy and coverage and a duration of action of only 2 years. There was no advantage to delaying their introduction as they act on an entirely different part of the parasite life cycle.

The modelling results from this study can be summarized as five major policy implications (listed in table 1). Although this model was developed specifically for Cambodia, these broad recommendations are relevant to malaria elimination efforts worldwide.

**Table 1:** Main policy implications of modelling results.

**Main policy implications:**
1. High coverage with ACT treatment can produce a long-term reduction in malaria whereas the impact of MDA is generally only short-term
2. Primaquine enhances the effect of ACT in eliminating malaria and reduces the increase in proportion of artemisinin resistant infections
3. Parasite prevalence is a better surveillance measure for elimination programmes than numbers of symptomatic cases
4. Combinations of interventions are most effective
5. Sustained efforts are crucial for successful elimination.

In conclusion, mathematical modelling when validated by good quality field data can combine information from diverse sources and be used as a tool for enhanced analysis to provide new insights into the results of clinical studies, to make predictions and to assist with planning future studies. This study has provided predictions and a number of novel insights which will be of direct practical benefit to assist planning of future malaria elimination strategies, particularly in the context of the newly emerging artemisinin resistance.

## Supporting Information

Figure S1 **Contribution of each component of the strategies employed in the field study to the reduction in the percent of the population with** ***P. falciparum*** **gametocytes.** Each panel shows the additional effect of adding primaquine to MDA with ACT with **A** MDA alone, **B** MDA combined with simultaneous introduction of ACT plus single PQ for treatment. Blue lines are treatment with ACT, red lines are treatment with ACT plus primaquine.
(TIF)

Figure S2 **Effect of varying the timing of interventions on the prevalence of parasitaemia in the population.** **A** MDA with ACT plus multiple primaquine and Rx with ACT plus single primaquine started together at different times after the start of 2004 (in months). **B** MDA with ACT plus multiple primaquine at different times after introducing Rx with ACT plus single primaquine in 2004 in months.
(TIF)

Supporting Information S1 **Summary equations.**
(DOCX)

Supporting Information S2 **Model fitting and validation with field data.**
(DOCX)

Supporting Information S3 **References for Supporting Information.**
(DOCX)

Table S1 **Assumptions.** The first 4 assumptions are likely to decrease the efficacy of interventions, whereas the next 5 assumptions probably increase the efficacy, for the reasons given. It is not known how the final assumption may affect the efficacy of interventions.
(DOCX)

Table S2 **Parameters.** Where possible, these were taken directly from the published field study data (‘Field study’) or by fitting model output to results of the field study (‘Fitting’). Other sources were unpublished interim reports for the field study (‘Report’), unpublished surveillance data from the Cambodia National Malaria Control Programme (CNM), discussion with the staff who ran the field study at CNM or co-authors for this manuscript (‘Verbal’). Parameters not specific to the field study were based largely on expert opinion of the co-authors and were derived from published data, where available, as stated below. For those parameters for which a range of values is given, this reflects uncertainty of their true value. For these parameters, the underlined values were used to generate the plots and results stated in the text and the ranges were used in the sensitivity analyses. For the efficacy of drug resistance on pharmacodynamics, as this is unknown, it was a modelled by multiplying the clearance rate for each drug by its relative effectiveness against resistant infections, ε, such that 0≤ε≤1.
(DOCX)

Table S3 **Results of fitting the model to field data to derive coverages of the different strategies employed in the field study.** One or both of prevalences of detected asexual parasitaemia and gametocytes were fitted as indicated. Strategy A was employed in 17 villages in Kampong Speu OD, B in 3 villages in Kampot OD and C in 4 villages in Kampong Speu OD.
(DOCX)
