# Supplementary figures and images for: Optimising Strategies for Plasmodium falciparum Malaria Elimination in Cambodia: Primaquine, Mass Drug Administration and Artemisinin Resistance
Source: PLoS One. 2012 May 25;7(5):e37166. doi: 10.1371/journal.pone.0037166 (PMC3360685; doi:10.1371/journal.pone.0037166)

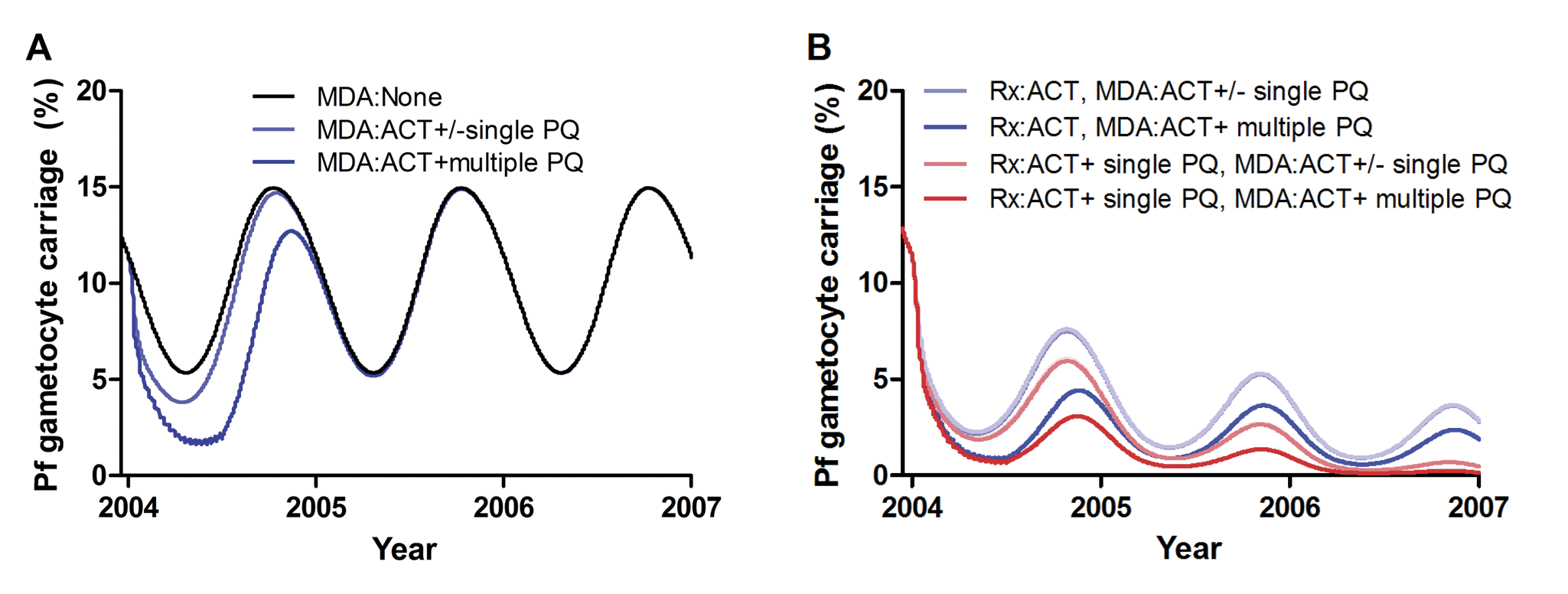

Supplement: Figure S1 — Contribution of each component of the strategies employed in the field study to the reduction in the percent of the population with P. falciparum gametocytes. Each panel shows the additional effect of adding primaquine to MDA with ACT with A MDA alone, B MDA combined with simultaneous introduction of ACT plus single PQ for treatment. Blue lines are treatment with ACT, red lines are treatment with ACT plus primaquine. (TIF) [file pone.0037166.s001.tif]

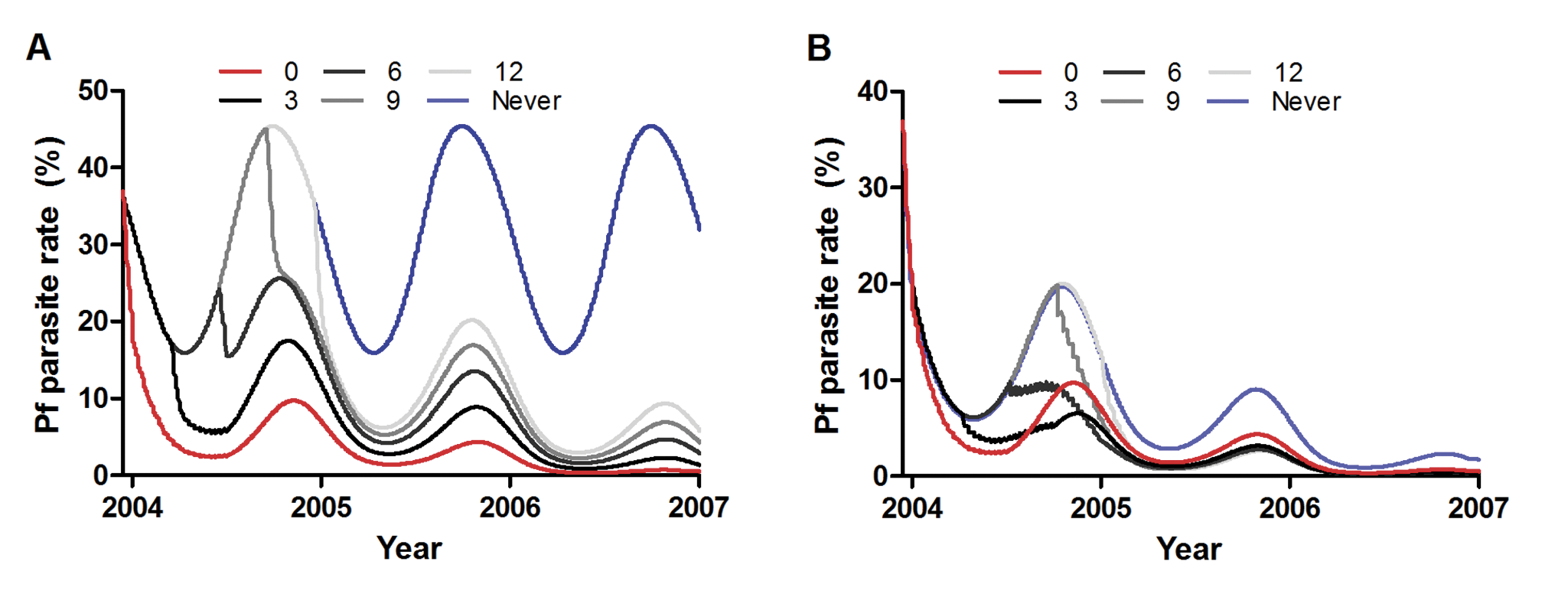

Supplement: Figure S2 — Effect of varying the timing of interventions on the prevalence of parasitaemia in the population. A MDA with ACT plus multiple primaquine and Rx with ACT plus single primaquine started together at different times after the start of 2004 (in months). B MDA with ACT plus multiple primaquine at different times after introducing Rx with ACT plus single primaquine in 2004 in months. (TIF) [file pone.0037166.s002.tif]
