# Supplementary material for: Optimising Strategies for Plasmodium falciparum Malaria Elimination in Cambodia: Primaquine, Mass Drug Administration and Artemisinin Resistance
Source: PLoS One. 2012 May 25;7(5):e37166. doi: 10.1371/journal.pone.0037166 (PMC3360685; doi:10.1371/journal.pone.0037166)
Supplement: Supporting Information S1 — Summary equations. (DOCX) [file pone.0037166.s003.docx]

**Summary Equations**

$$\dot{S}=bN- \frac{\beta S{(I}_{S}+I_{R})}{N}+p_{S}\left( v_{L}L_{S}+v_{B}B_{S}+v_{I}I_{S} \right)-\mu S+\delta I_{S}+wR$$

$$\dot{L_{S}=}\frac{\beta S{(I}_{S}+I_{R})}{N}-(\gamma+p_{S}v_{L}+\mu)L_{S}$$

$\dot{B_{S}=}\gamma L_{S}-(\sigma+p_{S}v_{B}+\mu)B_{S}$

$\dot{I_{S}=}\sigma B_{S}-\left( \delta+p_{S}v_{I}+\mu\right)I_{S}-yI_{S}$

$$\dot{R}=-\frac{\beta R{(I}_{S}+I_{R})}{N}+p_{R}\left( v_{L}L_{R}+v_{B}B_{R}+v_{I}I_{R} \right)-\mu R+dI_{R}+yI_{S}-wR$$

$$\dot{L_{R}=}\frac{\beta R{(I}_{S}+I_{R})}{N}-(\gamma+p_{R}v_{L}+\mu)L_{R}$$

$$\dot{B_{R}=}\gamma L_{R}-(\sigma+p_{R}v_{B}+\mu)B_{R}$$

$$\dot{I_{R}=}\sigma B_{R}-\left( d+p_{R}v_{I}+\mu\right)I_{R}$$

**Where:**

| $S$ | = susceptible, nonimmune |
| --- | --- |
| $L_{S}$ | = liver stage, nonimmune |
| $B_{S}$ | = blood stage non-infectious, nonimmune |
| $I_{S}$ | = blood stage infectious, nonimmune |
| $R$ | = susceptible, immune |
| $L_{R}$ | = liver stage, immune |
| $B_{R}$ | = blood stage non-infectious, immune |
| $I_{R}$ | = blood stage infectious, immune |

| $b$ | = birth rate |
| --- | --- |
| $\mu$ | = death rate |
| $\beta$ | = transmission parameter |
| $\gamma$ | = rate of going from liver stage to blood stage non-infectious |
| $\sigma$ | = rate of going from blood stage non-infectious to blood stage infectious |
| $\delta$ | = recovery rate in nonimmune population |
| $d$ | = recovery rate in immune population |
| $y$ | = rate of infectious blood stage in nonimmunes becoming immune |
| $w$ | = rate of losing immunity |
| $p_{S}$ | = proportion of nonimmunes who are symptomatic |
| $p_{R}$ | = proportion of immunes who are symptomatic |
| $v$ | = parasite clearance by drug action |
