# Supplementary material for: Optimising Strategies for Plasmodium falciparum Malaria Elimination in Cambodia: Primaquine, Mass Drug Administration and Artemisinin Resistance
Source: PLoS One. 2012 May 25;7(5):e37166. doi: 10.1371/journal.pone.0037166 (PMC3360685; doi:10.1371/journal.pone.0037166)
Supplement: Supporting Information S2 — Model fitting and validation with field data. (DOCX) [file pone.0037166.s004.docx]

**Model fitting and validation with field data**

**Surveillance data**

1. **Fitting to National surveillance data (2002-2004)**

In order to determine values for the timing and amplitude (amp and phi respectively) of seasonal variation in malaria prevalence in the study area, the model was fitted to baseline national surveillance data [32]. There was found to be a single annual peak in prevalence in mid-September and amplitude of variation of 0.55 of the peak prevalence. Root mean squared deviation (RMSD) for this fit was 7.38%.

1. **Fitting to Results of field study**

Although most details of the strategies used in the field study were recorded (7 and unpublished reports), it was not possible to measure the exact coverage for each component. The model was thus fitted to a range of field data from the field study to derive these coverages. Proportions of the population with detected asexual blood stage parasites and gametocytes were fitted simultaneously for strategies where these data were available.

The model was fitted to the results of three strategies investigated in the field study for which detailed results were available. The values for coverages for each component of these strategies were varied within realistic ranges (50-95%) set by discussion with the trial team until RMSD was minimised. Details of the strategies and results are shown in figures 2B-E and Table S3.

1. **Validation with Operational District surveillance data (2004-2010)**

The number of clinical cases from the model was validated against surveillance data from CNM for Kampot Operational District (OD) [unpublished] using an OD population (N0) of 122,330 [7] and trial population of 2387 [8]. During the trial period, the study team provided treatment with ACT plus primaquine to a much larger number of malaria cases in Kampot than were included in the trial (actual number not recorded). MDA was only used in the trial population. The high coverage with ACT plus single dose primaquine treatment likely resulted in the decrease in malaria cases seen throughout the OD during the trial period. The subsequent decreasing coverage following the departure of the study team probably resulted in the increase in cases from 2008 onwards. The model was able to reproduce the data using realistic values for coverage with the strategies employed in the field. The best fit was for a coverage with ACT plus single dose primaquine treatment of 60% of symptomatic cases during the trial, falling to 19% by 2008. RMSD for the best fit was 37.48 with the result shown in figure 2A.
