# Supplementary material for: Optimising Strategies for Plasmodium falciparum Malaria Elimination in Cambodia: Primaquine, Mass Drug Administration and Artemisinin Resistance
Source: PLoS One. 2012 May 25;7(5):e37166. doi: 10.1371/journal.pone.0037166 (PMC3360685; doi:10.1371/journal.pone.0037166)
Supplement: Supporting Information S3 — References for Supporting Information. (DOCX) [file pone.0037166.s005.docx]

**References for Supporting Information**

1. Maude RJ, Pontavornpinyo W, Saralamba S, Aguas R, Yeung S, et al. (2009) The last man standing is the most resistant: eliminating artemisinin-resistant malaria in Cambodia. Malar J 8: 31.
2. Walliker D, Hunt P, Hamza Babiker H (2005) Fitness of drug-resistant malaria parasites. Acta Trop 94: 251-259.
3. World Health Organization Regional Office for the Western Pacific (2007) Cambodia Health Situation and Trend 2007. Available: <http://www.wpro.who.int/NR/rdonlyres/BED34D62-D904-43B7-9CEC-ADA3CE991459/0/8Cambodia07.pdf>. Accessed 2011 June 29.
4. Yeung S, Van Damme W, Socheat D, White NJ, Mills A (2008) Access to artemisinin combination therapy for malaria in remote areas of Cambodia. Malaria J 7: 96.
5. World Health Organization (2010) Guidelines for the treatment of malaria second edition. Available: http://whqlibdoc.who.int/publications/2010/9789241547925_eng.pdf. Accessed 2012 February 27.
6. Anderson TJ, Nair S, Nkhoma S, Williams JT, Imwong M, et al. (2010) High heritability of malaria parasite clearance rate indicates a genetic basis for artemisinin resistance in western Cambodia. J Infect Dis 201: 1326-1330.
7. National Institute of Statistics of Cambodia, Ministry Of Planning (2004) Cambodia Inter-Censal Population Survey. Available: <http://statsnis.org/SURVEYS/depth-cips04/pro-cips/Table3_projection.htm>. Accessed 2011 June 29.
8. Song J, Socheat D, Tan B, Dara P, Deng C, et al. (2010) Rapid and effective malaria control in Cambodia through mass administration of artemisinin-piperaquine. Malar J 9: 57.
9. National Institute of Statistics of Cambodia, Ministry Of Planning (1998) First revision populations for Cambodia 1998-2020. Available: <http://statsnis.org/projcam/Provinfo_Proj.htm>. Accessed 2011 June 29.
10. Eyles DE, Young MD (1951) The duration of untreated or inadequately treated Plasmodium falciparum infections in the human host. J Natl Malar Soc 10: 327-336.
11. Collins WE, Jeffery GM (1999) A retrospective examination of sporozoite- and trophozoite-induced infections with Plasmodium falciparum: development of parasitologic and clinical immunity during primary infection. Am J Trop Med Hyg 61: 4-19.
12. Kitchen SF (1949) Falciparum Malaria. In Malariology. Philadelphia: W.B. Saunders Co. 995-1016 p.
13. Franks S, Koram KA, Wagner GE, Tetteh K, McGuinness D (2001) Frequent and persistent, asymptomatic Plasmodium falciparum infections in African infants, characterized by multilocus genotyping. J Infect Dis 183: 796-804.
14. Bruce MC, Donnelly CA, Packer M, Lagog M, Gobson N (2000) Age- and species-specific duration of infection in asymptomatic malaria infections in Papua New Guinea. Parasitology 121: 247-256.
15. Babiker HA, Abdel-Muhsin AM, Ranford-Cartwright LC, Satti G, Walliker D (1998) Characteristics of Plasmodium falciparum parasites that survive the lengthy dry season in eastern Sudan where malaria transmission in markedly seasonal. Am J Trop Med Hyg 59: 582-590.
16. Thompson D (1911) A research into the production, life and death of crescents in malignant tertian malaria, in treated and untreated cases by an enumerative method. Ann Trop Med Parasitol 5: 57-85.
17. Jeffrey GM, Eyles DE (1955) Infectivity to mosquitoes of Plasmodium falciparum as related to gametocyte density and duration of infection. Am J Trop Med Hyg 4: 781-789.
18. Aguas R, Lourenço JM, Gomes MG, White LJ (2009) The impact of IPTi and IPTc interventions on malaria clinical burden - in silico perspectives. PLoS One 4: e6627.
19. White NJ (1997) Assessment of the pharmacodynamic properties of antimalarial drugs in vivo. Antimicrob Ag Chemother 41: 1413-1422.
20. [Karbwang J](http://www.ncbi.nlm.nih.gov/sites/entrez?Db=pubmed&Cmd=Search&Term=%22Karbwang%20J%22%5BAuthor%5D&itool=EntrezSystem2.PEntrez.Pubmed.Pubmed_ResultsPanel.Pubmed_DiscoveryPanel.Pubmed_RVAbstractPlus), [Na-Bangchang K](http://www.ncbi.nlm.nih.gov/sites/entrez?Db=pubmed&Cmd=Search&Term=%22Na-Bangchang%20K%22%5BAuthor%5D&itool=EntrezSystem2.PEntrez.Pubmed.Pubmed_ResultsPanel.Pubmed_DiscoveryPanel.Pubmed_RVAbstractPlus), [Congpoung K](http://www.ncbi.nlm.nih.gov/sites/entrez?Db=pubmed&Cmd=Search&Term=%22Congpoung%20K%22%5BAuthor%5D&itool=EntrezSystem2.PEntrez.Pubmed.Pubmed_ResultsPanel.Pubmed_DiscoveryPanel.Pubmed_RVAbstractPlus), [Thanavibul A](http://www.ncbi.nlm.nih.gov/sites/entrez?Db=pubmed&Cmd=Search&Term=%22Thanavibul%20A%22%5BAuthor%5D&itool=EntrezSystem2.PEntrez.Pubmed.Pubmed_ResultsPanel.Pubmed_DiscoveryPanel.Pubmed_RVAbstractPlus), [Harinasuta T](http://www.ncbi.nlm.nih.gov/sites/entrez?Db=pubmed&Cmd=Search&Term=%22Harinasuta%20T%22%5BAuthor%5D&itool=EntrezSystem2.PEntrez.Pubmed.Pubmed_ResultsPanel.Pubmed_DiscoveryPanel.Pubmed_RVAbstractPlus) (1998) Pharmacokinetics of oral artesunate in thai patients with uncomplicated falciparum malaria. Clin Drug Investig 15: 37-43.
21. Tarning J, Ashley EA, Lindegardh N, Stepniewska K, Phaiphun L, et al. (2008) Population pharmacokinetics of piperaquine after two different treatment regimens with dihydroartemisinin-piperaquine in patients with Plasmodium falciparum malaria in Thailand. Antimicrob Ag Chemother 52: 1052-1061.
22. Burges RW, Bray RS (1961) The effect of a single dose of primaquine on the gametocytes, gametogony and sporogony of Laverania falciparum. Bull World Health Organ 24: 451-456.
23. International Artemisinin Study Group (2004) Artesunate combinations for treatment of malaria: meta-analysis. Lancet 363: 9-17.
24. Myinta HY, Ashley EA, Day NJP, Nosten F, White NJ (2007) Efficacy and safety of dihydroartemisinin-piperaquine. Trans R Soc Trop Med Hyg 101: 858-866.
25. Janssens B, van Herp M, Goubert L, Chan S, Uong S, et al. (2007) A randomized open study to assess the efficacy and tolerability of dihydroartemisinin-piperaquine for the treatment of uncomplicated falciparum malaria in Cambodia. Trop Med Int Health 12: 251-259.
26. Chen L, Qu FY, Zhou YC (1982) Field observations on the antimalarial piperaquine. Chin Med J 95: 281-286.
27. Rieckman KH, McNamara JV, Kass L, Powell RD (1969) Gametocytocidal and Sportontocidal Effects of Primaquine Upon Two Strains of Plasmodium Falciparum. Mil Med 134: 802-819.
28. Smithuis F, Kyaw MK, Phe O, Win T, Aung PP, et al. (2010) Effectiveness of five artemisinin combination regimens with or without primaquine in uncomplicated falciparum malaria: an open-label randomised trial. Lancet Infect Dis 10: 673-681.
29. Dondorp AM, Nosten F, Yi P, Das D, Phyo AP, et al. (2009) Artemisinin resistance in Plasmodium falciparum malaria. N Engl J Med 361: 455-67.
30. Dolan G, ter Kuile FO, Jacoutot V, White NJ, Luxemburger C, et al. (1993) Bed nets for the prevention of malaria and anaemia in pregnancy. Trans R Soc Trop Med Hyg 87: 620-626 .
31. Sochantha T, Hewitt S, Nguon C, Okell L, Alexander N, et al. (2006) Insecticide-treated bednets for the prevention of Plasmodium falciparum malaria in Cambodia: a cluster-randomized trial. Trop Med Int Health 11: 1166-1177.
32. National Center for Parasitology, Entomology and Malaria Control, Ministry of Health, Cambodia (2007) Annual progress report of the National Center for Parasitology, Entomology and Malaria Control. 26-39 p.
