# Supplementary material for: Optimising Strategies for Plasmodium falciparum Malaria Elimination in Cambodia: Primaquine, Mass Drug Administration and Artemisinin Resistance
Source: PLoS One. 2012 May 25;7(5):e37166. doi: 10.1371/journal.pone.0037166 (PMC3360685; doi:10.1371/journal.pone.0037166)
Supplement: Table S1 — Assumptions. The first 4 assumptions are likely to decrease the efficacy of interventions, whereas the next 5 assumptions probably increase the efficacy, for the reasons given. It is not known how the final assumption may affect the efficacy of interventions. (DOCX) [file pone.0037166.s006.docx]

|  | **Assumption** | **Reasoning for likely effect on efficacy of interventions if assumption is true** | **Justification for making the assumption** |
| --- | --- | --- | --- |
| **Likely to *decrease* efficacy of interventions** | No survival disadvantage (fitness cost) for artemisinin resistant parasites compared to drug sensitive parasites. | More robust resistant parasites are harder to eradicate. Fitness cost reduces the rate of spread of artemsinin resistance and increases the effectiveness of an intervention against resistant parasites [1]. | The relative viability of malaria parasites with artesunate resistant phenotypes is not known [2]. Regardless, it should have minimal impact on the effectiveness of interventions when resistance is rare or absent, as in this field study. |
|  | No mortality due to malaria. | Those people with resistant infections are less likely to respond to treatment and therefore more likely to die, thus removing them from the transmitting population. | The proportion of malaria infections which are fatal in this region is around 0.6% [3], a small proportion of overall infections. |
|  | Artesunate monotherapy was the only available effective treatment before 2004. | Artesunate would be less likely to cure artemisinin-resistant infections than non-artemisinin drugs, if available. If significant amounts of ACT were available, this would decrease the baseline parasite prevalence. | Although co-blistered artesunate and mefloquine has been the official first-line drug since 2000, in reality a wide range of treatments is available over the counter in Cambodia. At the time of the field study, many received artesunate monotherapy and most of the other treatments were inadequate (resistant parasites/wrong dose/wrong duration) to cure infection [4]. |
|  | No treatment of nonmalarial fever with antimalarials. | Some people with incidental asymptomatic malaria infection would also receive treatment. | Presumptive treatment of malaria is not standard practice in Cambodia and is discouraged worldwide [5]. |
|  |  |  | There were no data available with which to parameterise such a model for Cambodia.  To maintain simplicity. |
|  |  |  |  |
| **Likely to *increase* efficacy of interventions** | Rate of resistance to artesunate is very low or absent at the time of the trial. | If the rate of resistance was high then interventions including ACT would be less effective. This is explored elsewhere in this paper. | Expert opinion in the absence of data. |
|  | No resistance primaquine. | If resistance was present then interventions including primaquine would be less effective. | No primaquine resistance has been identified in Cambodia. |
|  | No spatial heterogeneity | Infection in high transmission areas is harder to eradicate therefore taking longer. | A non-spatial framework was chosen to maintain flexibility and efficiency to rapidly explore a wide range of scenarios in accordance with the aims of this study. |
|  |  |  | In the absence of data about many spatially heterogeneous parameters (seasonal variation in parasite prevalence, coverage with interventions, geographical extent of each village, etc.) we felt their incorporation at this stage was premature. |
|  |  |  | We are in the ongoing process of gathering detailed data to develop a spatial model based on the framework presented here. |
|  | No population migration. | People do not continue to introduce new resistant parasites. | To maintain simplicity. |
|  |  |  | Although studies are underway, there is currently insufficient data to parameterize the model for migration. |
|  |  |  | In-migration of infections would slow the reduction of malaria, possibly precluding its elimination. Out-migration of infections would do the opposite. |
|  | No stochasticity | Infections more likely to spontaneously eliminate when numbers are small. | A deterministic framework was chosen to maintain flexibility and efficiency to rapidly explore a wide range of scenarios in accordance with the aims of this study. |
|  | Single infecting clone in each individual | The mixing of multiple clones within a host may aid in the selection of fitter parasites through competition for resources. This would reduce the likelihood of elimination. | To maintain simplicity. A model of multiple clones within a host would be far more complex than that presented here. |
|  |  | Alternatively, interbreeding between clones may dilute any drug resistance mutations in the population and thus increase the likelihood of elimination. | Most infections in Cambodia are with a single clone [6], although multiple clones are common in high transmission settings. |
|  |  |  | It is not understood how multiple clones interact within a host and how this affects the transmission dynamics. |
