# Supplementary material for: Optimising Strategies for Plasmodium falciparum Malaria Elimination in Cambodia: Primaquine, Mass Drug Administration and Artemisinin Resistance
Source: PLoS One. 2012 May 25;7(5):e37166. doi: 10.1371/journal.pone.0037166 (PMC3360685; doi:10.1371/journal.pone.0037166)
Supplement: Table S2 — Parameters. Where possible, these were taken directly from the published field study data (‘Field study’) or by fitting model output to results of the field study (‘Fitting’). Other sources were unpublished interim reports for the field study (‘Report’), unpublished surveillance data from the Cambodia National Malaria Control Programme (CNM), discussion with the staff who ran the field study at CNM or co-authors for this manuscript (‘Verbal’). Parameters not specific to the field study were based largely on expert opinion of the co-authors and were derived from published data, where available, as stated below. For those parameters for which a range of values is given, this reflects uncertainty of their true value. For these parameters, the underlined values were used to generate the plots and results stated in the text and the ranges were used in the sensitivity analyses. For the efficacy of drug resistance on pharmacodynamics, as this is unknown, it was a modelled by multiplying the clearance rate for each drug by its relative effectiveness against resistant infections, ε, such that 0≤ε≤1. (DOCX) [file pone.0037166.s007.docx]

|  | **Symbol** | **Description** | **Value** | **Source** |
| --- | --- | --- | --- | --- |
| **Population demographics** | N_0_ | Population of Kampot Operational District | 122,330 | [7] |
|  |  | Overall study population | 3,653 | [8] |
|  | μ | Birth rate = death rate | 15/1000/year | [7,9] |
| **Prevalence of malaria in population** | p_inf_ | Proportion of population with blood stage infection when interventions start | 0.5585 | Fitting |
|  | p_a_ | Proportion of malaria infections that were resistant to artesunate in 2004 | 0,5,10-80% | Expert opinion |
|  | p_b_ | Proportion of malaria infections that were resistant to piperaquine in 2004 | 0.05 | Expert opinion |
| **Natural history of malaria infection** | δ | Recovery rate from untreated infectious blood stage infection (nonimmune) | 1/200 - 1/60 days^-1^ | [10-15] |
|  | δ_R_ | Recovery rate from untreated infection (immune) | 1/200 - 1/30 days^-1^ | [10-15] |
|  | Γ | Rate of liver stage becoming blood stage | 1/5 days^-1^ | [10-12] |
|  | σ | Rate of blood stage becoming gametocytes | 1/15 days^-1^ | [16,17] |
|  | amp | Amplitude of seasonal variation of transmission | 0.55 | Fitting |
|  | phi | Peak of seasonal variation of transmission | 0.62 | Fitting |
|  | Ω | Duration of immunity to malaria | 0.5, 1-5 years | [18] |
|  | propRx_R_ | Proportion of infected cases with immunity who are treated | 0-0.1-1.0 | [18] |
|  | propRx_N_ | Proportion of infected cases without immunity who are treated | 0.9 | [18] |
| **Artemisinin monotherapy** | start_a_ | Year of introduction of artemisinin monotherapy | 1975 | Expert opinion |
|  | ԏ = ԏ_ai1_  = ԏ_ai2_ | Rate of starting artemisinin monotherapy | 1/16 infected people per day | [4] |
|  | propRx_am_ | Proportion of infected population who receive antimalarials | 0.63 | [4] |
|  | prop_a_ | Proportion of antimalarials constituting artemisinin monotherapy before a intervention | 0.4 | [4] |
|  | adh_a_ | Proportion of infected population that take full 7 day course of artemisinin monotherapy | 0.2 | [4] |
|  | propRx_a_ | Proportion of infected population that take effective artemisinin monotherapy = propRxam*propa*adha | 0.05 | = PropRx_am_*prop_a_ *adh_a_ |
| **Interventions** | ԏ_ab_ = ԏ_ab1_  = ԏ_ab2_ | Rate of reaching maximum coverage with ACT treatment | 1/14 days^-1^ | [4] |
|  | ԏ_MDA1_  = ԏ_MDA2_ | Rate of reaching maximum coverage with MDA | 1/14 days^-1^ | Verbal/Report |
|  | cov_MDA1_ | Maximum coverage of first MDA | 0, 0.51 or 0.95 | Fitting |
|  | cov_MDA2_ | Maximum coverage of second MDA | 0 or 0.95 | Fitting |
|  | T_MDA2_ | Time to start of second MDA | 42 or 365 days | Trial |
|  | dur_i1_ = dur_i2_ | Duration of single round of MDA | 14 days | Verbal |
|  | dur_PP_ | Duration of multiple rounds of primaquine MDA | 6 months | Trial |
|  | freq_PP_ | Frequency of multiple rounds of primaquine MDA | 1/10 days^-1^ | Trial |
|  | cov_ab_ | Coverage with ACT treatment during trial | 0.78 or 0.95 | Fitting |
|  | cov_ab2_ | Coverage with ACT treatment after trial | 0.19 | Fitting |
|  | p_sab_ | Proportion of vendors selling modern drugs that could sell ACT | 0.85 | [4] |
|  | adh_ab_ | Adherence to 2 day course of ACT | 0.77 | [4] |
|  | propRx_i1_  = propRx_i2_ | Proportion that receive full 2 day course of MDA | 0.73 | = cov_i1_*adh_ab_  or cov_i2_*adh_vg_ |
|  | p_ab_ | Proportion that actually receive full 2 day course of ACT during field study | 0.62 | = cov_ab_*p_sab_*adh_ab_ |
|  | sens_Dx_ | Sensitivity of diagnostic test used to decide whether to treat febrile cases | 0.8 | Expert opinion |
|  | dur_1_ = dur_2_ | Total duration of MDA | 0 or 6 months | Trial |
|  | dur_a_ | Duration of availability of artemisinin monotherapy | 0 years or long-term | Expert opinion |
|  | dur_ab_ | Duration of availability of ACT | 0 years or long-term | Expert opinion |
| **Duration of efficacy against sensitive parasites (**$\mathbf{X}$**)** | X_ao_ | Full course of artemisinin monotherapy | 7 days | [19,20] |
|  | X_ai_ | Artemisinin as part of ACT (2 day course) | 2 days | [19] |
|  | X_b_ | Piperaquine | 20-30 days | [21] |
|  | X_p_ | Primaquine (1 day course) | 1 day | [22] |
| **Rates of clearance of drug sensitive infection (**$\nu$) **by treatment** | c_Broda_ | Artemisinin vs non-infectious blood stage | 1/7 days^-1^ | [23] |
|  | c_Iroda_ | Artemisinin vs infectious blood stage | 1/4days^-1^ | [1] |
|  | C_Irodab,_ C_Brodab_ | Artemether-piperaquine vs infectious or non-infectious blood stage | 1/3 days^-1^ | [23,24,25] |
|  | c_Brodb_ | Piperaquine vs non-infectious blood stage | 1/3 days^-1^ | [26] |
|  | c_Irodb_ | Piperaquine vs infectious blood stage | 1/21 days^-1^ | [24] |
|  | c_Ldp_ | Primaquine vs liver stage infection (9mg) | 1/14-1/7days^-1^ | [22] |
|  | c_Idp_ | Primaquine vs infectious blood stage infection (9mg) | 1/4, 1/2, 1/1 days^-1^ | [22,27,28] |
| **Effect of drug resistance on pharmacodynamics** | pct_roda_ | Parasite clearance time for artemisinin vs sensitive infections | 30 hours | [29] |
|  | pct_rada_ | Parasite clearance time for artemisinin vs resistant infections | 83 hours | [29] |
|  | p_recra_ | Proportion of infections resistant to artemisinin that recrudesce after treatment with artemisinin monotherapy | 0.35 | [29] |
|  | ε_rada_ | Relative effectiveness of artemisinin against artemisinin resistant parasites | 0.27 | = pct_roda_/pct_rada_ *(1-p_recra_) |
|  | ε_rbdb_ | Relative effectiveness of piperaquine against resistant parasites | 0.8 | [26] |
| **Bed nets** | $\rho$ | Degree of transmission reduction (the product of coverage and efficacy) | 0.3 | [30,31] |
|  | tau_bn_ | Time to introduce bed nets | 1 month | Expert opinion |
|  | cov_bn_ | Coverage with insecticide treated bed nets | 0 to 0.75 | Expert opinion |
|  | dur_bn_ | Duration of effectiveness of bed nets | 0 or 2 years | Expert opinion |
