# Supplementary material for: Optimising Strategies for Plasmodium falciparum Malaria Elimination in Cambodia: Primaquine, Mass Drug Administration and Artemisinin Resistance
Source: PLoS One. 2012 May 25;7(5):e37166. doi: 10.1371/journal.pone.0037166 (PMC3360685; doi:10.1371/journal.pone.0037166)
Supplement: Table S3 — Results of fitting the model to field data to derive coverages of the different strategies employed in the field study. One or both of prevalences of detected asexual parasitaemia and gametocytes were fitted as indicated. Strategy A was employed in 17 villages in Kampong Speu OD, B in 3 villages in Kampot OD and C in 4 villages in Kampong Speu OD. (DOCX) [file pone.0037166.s008.docx]

| Strategy | Components | Derived coverage (%) | Fitted output | RMSD (%) |
| --- | --- | --- | --- | --- |
| A | 1. MDA ACT+PP | 94.99 | Asexual | 0.70 |
|  | 2. Rx ACT+P | 78.08 | Gametocytes | 0.29 |
|  |  |  | Asexual+gametocytes | 1.16 |
| B | 1. MDA ACT+PP | 94.95 | Asexual | 5.97 |
|  | 2. Rx ACT+P | 92.45 |  |  |
|  | 3. MDA2 ACT+P at 42 days | 95.0 |  |  |
| C | 1. MDA ACT+PP | 51.03 | Asexual | 7.12 |
|  | 2. Rx ACT+P | 94.99 |  |  |
|  | 3. MDA2 ACT+P at 1 year | 94.9 |  |  |
